# Aging and Autophagy: Roles in Musculoskeletal System Injury

**DOI:** 10.14336/AD.2024.0362

**Published:** 2025-06-03

**Authors:** Haifeng Zhang, Wenhui Gu, Genbin Wu, Yinxian Yu

**Affiliations:** ^1^Department of Orthopedics Surgery, Shanghai General Hospital, Shanghai Jiao Tong University School of Medicine, Shanghai, China.; ^2^Department of Physiology and Hypoxic Biomedicine, Institute of Special Environmental Medicine, Nantong University, Nantong, Jiangsu, China.

**Keywords:** aging, senescence, autophagy, musculoskeletal system

## Abstract

Aging is a multifactorial process that ultimately leads to a decline in physiological function and a consequent reduction in the health span, and quality of life in elderly population. In musculoskeletal diseases, aging is often associated with a gradual loss of skeletal muscle mass and strength, resulting in reduced functional capacity and an increased risk of chronic metabolic diseases, leading to impaired function and increased mortality. Autophagy is a highly conserved physiological process by which cells, under the regulation of autophagy-related genes, degrade their own organelles and large molecules by lysosomal degradation. This process is unique to eukaryotic cells and is a strict regulator of homeostasis, the maintenance of energy and substance balance. Autophagy plays an important role in a wide range of physiological and pathological processes such as cell homeostasis, aging, immunity, tumorigenesis and neurodegenerative diseases. On the one hand, under mild stress conditions, autophagy mediates the restoration of homeostasis and proliferation, reduction of the rate of aging and delay of the aging process. On the other hand, under more intense stress conditions, an inadequate suppression of autophagy can lead to cellular aging. Conversely, autophagy activity decreases during aging. Due to the interrelationship between aging and autophagy, limited literature exists on this topic. Therefore, the objective of this review is to summarize the current concepts on aging and autophagy in the musculoskeletal system. The aim is to better understand the mechanisms of age-related changes in bone, joint and muscle, as well as the interaction relationship between autophagy and aging. Its goal is to provide a comprehensive perspective for the improvement of diseases of the musculoskeletal system.

## 1. Introduction

Aging refers to the inevitable functional deterioration, internal instability, reduced resilience and progressive breakdown of the structure and components of the physiological organs of an organism with age, which leads to irreversible death [1]. It is also one of the major risk factors for human diseases including cancer, cardiovascular disease, diabetes and neurodegenerative diseases. Approximately 150,000 people die each day worldwide, with two-thirds of these deaths attributable to aging [2]. Almost all multicellular organisms show signs of aging over time. The gradual loss or deterioration of tissue and organ function results in many chronic age-related diseases. The underlying basis for these conditions may be shared by some common molecular and cellular mechanisms. Cell senescence is a response of cells to stress that involves irreversible cell cycle arrest and loss of proliferative activity [3]. The phenomenon of cellular senescence was first observed in 1961 by the microbiologists Leonard Hayflick and Paul Moorhead, who observed that fibroblasts in vitro cultures stopped growing after more than 50 passages [4]. Cellular senescence is a prominent manifestation of the aging process and is a driving factor in the development of many age-related diseases. In addition, cellular senescence can occur at any stage of life, from the embryonic stage to adulthood [5-6]. It is a highly regulated process, and when any part of this process is disrupted, it can have both positive effects on the body, such as playing an essential role in normal development, maintaining tissue homeostasis, and inhibiting tumor proliferation, as well as negative effects, driving age-related pathological processes [7-8]. Understanding the mechanisms of aging, its effects on organ function, and the prevention or slowing of aging is therefore of paramount importance [9].

**Figure 1.**
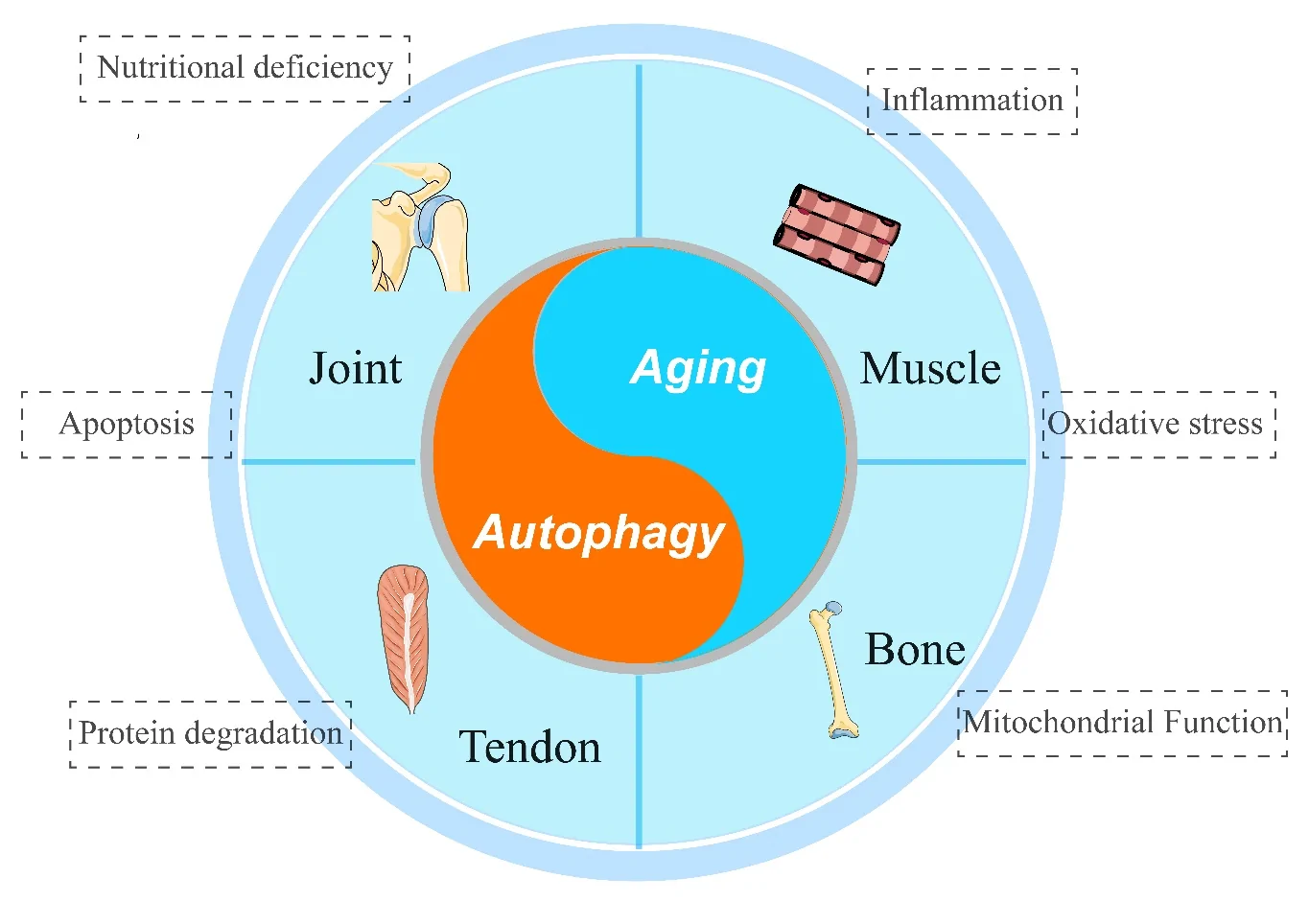
Search for injuries to the musculoskeletal system on the topic of autophagy and aging, including the mechanisms of injury that occur in the bones, muscles, tendons, and joints.

This review focuses on aspects of musculoskeletal injuries, summarized with aging and autophagy as the main keywords. The Pubmed and Web of Science websites were then searched using keywords such as aging, autophagy, and musculoskeletal system, and the main scope of the review is shown in Figure 1.

## 2. Aging involved various physiological mechanisms

The current point of view is that cellular senescence is a self-protective mechanism of the cells in response to endogenous and exogenous stress. It occurs under conditions including DNA damage, telomere shortening, oncogene activation, epigenetic changes, oxidative stress, and others [10]. It is characterized by permanent growth arrest, where the cell cycle stops at G1 or S to prevent the propagation of abnormal genes to the next generation of cells, thereby maintaining organismal homeostasis [11-12]. During cellular senescence, cells undergo changes in their morphology, which typically include enlargement and flattening [13]. Depending on the cause of senescence, it can be classified into replicative senescence and stress-induced premature senescence [14], the latter including various types such as DNA damage senescence, oxidative stress senescence, epigenetic senescence, and inflammatory senescence. Increased expression of the p21, p16 and p53 pathways, enhanced activity of senescence-associated β-galactosidase (SA-β-gal) and high levels of reactive oxygen species (ROS) are typical features of stress-induced premature senescence [15]. In addition, a high level of expression of p19ARF, p53, and plasminogen activator inhibitor-1 (PAI-1) has been observed in senescent cells and has been used as a marker for senescent cells [16]. Although senescent cells stop dividing, they still possess metabolic functions and can secrete a variety of senescence-associated secretory phenotypes (SASP), including matrix metalloproteinases (MMPs), growth factors, pro-inflammatory cytokines, and chemokines. These secretions act through paracrine pathways to increase local expression levels of inflammatory mediators and matrix-degrading enzymes, thereby affecting surrounding cells and tissues [17]. Acute cellular senescence contributes to the maintenance of organismal homeostasis. Secreted growth factors can promote embryonic development [18-19], wound healing [20], and insulin secretion [21]. However, the persistence of senescent cells accelerates the dysfunction of tissues and organs, leading to the degenerative diseases associated with aging [22].

Cellular senescence is a key mechanism in the pathogenesis of age-related diseases. With increasing age, senescent cells accumulate in a variety of organs and systems and are accompanied by inflammation, oxidative stress, and cell apoptosis [23]. The actual age of an individual is different from his or her biological state of aging [24]. In studies by Giuseppe et al, the number of circulating CD34^+^ cells have been proposed to be a predictor of life span in the oldest individuals, under the influence of apoptosis, oxidative stress, telomere shortening and inflammation [25]. DNA methylation age is an epigenetic biomarker of aging, more accurate than chronological age in predicting disease incidence and aging status [26]. Aging leads to a decline in a variety of bodily functions, including immune function [27], and is associated with a number of diseases such as cancer, Alzheimer’s disease, and a number of musculoskeletal disorders [28]. In the musculoskeletal system in particular, age is manifested in reduced bone mass and microstructural damage. In sarcopenia, where muscle mass and function decline with age, age-related inflammation is a major pathogenic factor [29]. In addition, it contributes to the degeneration of intervertebral discs, senile osteoporosis, and arthritis and tendon disorders caused by the aging of the tendons [30-33].

## 3. The Physiological Regulation of Autophagy in the Body

Autophagy is a cellular process that involves the engulfment of cellular components and their delivery to the lysosomes for degradation [34]. It plays a regenerative role by recycling and reusing parts of the cell, thereby maintaining the internal stability of the environment and providing a timely response to damaged and aging cells [35]. When autophagy occurs, autophagosomes and lysosomes fuse normally. However, when autophagic flux is inhibited, it reduces the ability to remove cellular waste and exacerbates cell death [36].

Autophagy is initiated by a number of autophagy-related genes. These are mainly classified into macroautophagy. microautophagy, chaperone-mediated autophagy. and selective autophagy [37], Macro-autophagy can be further subdivided into mitophagy, nucleophagy, pexophagy, aggregophagy, and xenophagy [38-39], Tlie most typical and common form of autophagy is microautophagy. while selective autophagy selectively degrades specific materials such as aging organelles (e.g.. mitochondria, endoplasmic reticulum), thereby regulating organelle function. During nutrient deprivation, autophagy can promote cell survival, but excessive activation of the autophagic mechanism can be a cause of cell death [40], Iron starvation can induce autophagy, as proposed in the study by Liu et al. Transmembrane protein 164 mediates the induction of autophagy. leading to iron accumulation and promoting iron-dependent cell death, a form of autophagy-dependent death [41], Autophagy can also be induced when exposed to genetic toxic stress [42]. LC3-associated phagocytosis (LAP) and pore-fonnmg toxin-induced non-canonical autophagy (PINCA) are both noncanonical autophagy pathways [42], These pathways are capable of modification of single membrane vesicles with ATG8 family proteins. To enhance macrophage activity against Listeria, Listeria-containmg phagosomes can be targeted by this non-canonical autophagy pathway. However, non-canonical autophagy mduced by the pore-forming toxin does not have a clear antimicrobial function. However, it may play a role in the repair of damaged vesicle membranes. Autophagy regulates the body primarily by modulating inflammatory responses and stress resistance, regulating mitochondrial function, and degrading abnormal proteins. In domg so, it maintains environmental homeostasis [43].

### 3.1 Regulating Inflammation

Decreased cellular autophagy can lead to lysosomal permeabilization, thereby triggering inflammation [44]. Dai et al. found that reduced cellular autophagic capacity promotes the release of inflammatory factors, impairs the function of osteoblasts, and facilitates inflammation in osteoarthritis [45]. In addition, Leena P et al. found that metformin may have an anti-inflammatory effect through an increase in mitochondrial autophagy [46]. In a pulmonary inflammation study, balanced polarization between M1 and M2 macrophages was observed to regulate lung inflammation, with M1 macrophages, typically associated with autophagy inhibition, promoting inflammation, while M2 macrophages, with anti-inflammatory properties, can be polarized via autophagy to ameliorate inflammation [47-48].

### 3.2 Anti-stress Response

Under conditions of hypoxia or oxidative stress, autophagy can be a cell survival enhancer [49]. In cancer therapy, induction of autophagy in tumor cells can increase oxidative stress-induced damage, which can lead to tumor cell death. Mfn2, an outer mitochondrial membrane protein, is involved in the regulation of mitochondrial function. Its over-expression induces mitochondrial autophagy, which requires the involvement of reactive oxygen species (ROS), since ROS can activate the PINK1/Parkin signaling pathway and thereby trigger mitochondrial autophagy [50]. Reducing the number of mitochondria helps counteract oxidative stress because mitochondria are the primary site of intracellular ROS generation [51].

### 3.3 Regulating Mitochondrial Function

Autophagy can regulate the function of organelles, in particular that of the mitochondria. Lysosomes are critical for autophagy, and their dysfunction can cause cellular iron depletion and subsequent mitochondrial dysfunction [52]. Furthermore, proteins involved in mitochondrial autophagy can recruit autophagic factors to damaged mitochondria to promote degradation [53]. Studies in lung fibrosis have shown that BMP4, a multifunctional growth factor, promotes selective mitochondrial autophagy in lung fibroblasts, which restores the balance of mitochondrial dynamics. This prevents sustained activation of lung fibroblasts and improves lung function [54]. Another study suggested that the promotion of mitochondrial autophagy should be considered as a target to counteract cerebral hemorrhage, which is associated with impaired mitochondrial function [55-56].

### 3.4 Degradation of Abnormal Proteins

In addition to the transport of organelles for degradation within lysosomes, autophagy also plays a similar role in protein degradation and serves as the primary pathway for the turnover of stable and defective proteins within cells [57]. The AGT8 family of proteins has been implicated in autophagy. In a related study, interactions between six homologous human proteins and 67 other proteins were observed, revealing a large degree of overlap between the members of the family and their potential functions [58]. Protein aggregation has been implicated in the pathogenesis of various diseases. The chaperone protein subunit CCT2 functions as an autophagic receptor, facilitating the clearance of aggregating proteins and enhancing the autophagic degradation of less mobile protein aggregates [59]. In addition, autophagy has been implicated in the degradation of abnormal proteins and the maintenance of environmental stability. One study showed that exercise increased autophagosome formation or decreased autophagosome clearance in middle-aged and elderly sprinters, which is beneficial for maintaining muscle protein homeostasis [60-61].

## 4. The crosstalk between autophagy and aging

Cell senescence can be induced by a variety of external and internal stresses through two main mechanisms, namely the activation of the p53-p21 signaling pathway and/or the activation of the p16INK4a-Rb signaling pathway [62]. Examples of these stresses include telomeric attrition, DNA damage, oxidative stress, mitochondrial dysfunction, and aberrant levels of cellular autophagy [63-64]. Research indicates that decreased levels of cellular autophagy, leading to reduced degradation capacity of damaged organelles and proteins, are often associated with the process of cellular senescence. In addition, reduced levels of cellular autophagy accelerate the aging process. On the other hand, moderately increased levels of autophagy have an anti-aging effect [65]. In mammalian organisms, cellular autophagy is a highly conserved catabolic process. Under stress conditions, such as nutrient deprivation and hypoxia, autophagy is activated. It is finely regulated by the assembly of multiple protein complexes. As a key regulator of autophagy, Beclin-1 is an essential component of the class III PI3K complex required for autophagosome formation [66]. The level of the Beclin-1 protein influences its activity and thus determines, to a certain extent, the level of cellular autophagy [67]. Beclin-1-dependent autophagy is an essential cellular pathway for the extension of the life span of organisms. In addition, Beclin-1 contains a BH3 domain. Through this domain, the anti-apoptotic factor BCL-2 binds to Beclin-1, affecting its activity and suppressing the level of cellular autophagy. Recent studies have confirmed that basal autophagic levels in tissues can be increased, organism lifespan prolonged and cell senescence reversed by inhibiting BCL-2 or interfering with its binding to Beclin-1 [63]. In the meantime, it has also been shown that Beclin-1-dependent cellular autophagy plays an important role in certain age-related degenerative diseases under pathological conditions [68]. In the field of pulmonary fibrosis, numerous studies have shown that autophagy is insufficient in alveolar epithelial cells of IPF (idiopathic pulmonary fibrosis), and that knockout of autophagy-related proteins leads to abnormalities in lamellar bodies and increased endoplasmic reticulum stress, thereby exacerbating cell senescence [69-70]. A down-regulation of the expression of Beclin-1 has also been observed in the fibrotic cells of IPF. In addition, research has shown that Beclin-1-dependent autophagy is involved in the regulation of the anti-fibrotic effects of nintedanib [71-72].

Autophagy is an important cellular quality control mechanism that participates in the degradation and renewal of mitochondria. This improves mitochondrial function and muscle health [73]. The interaction between autophagy and aging is very close. Autophagy plays a crucial role in the removal of cellular waste products and damaged organelles, thereby contributing to the delay of the aging process. Conversely, the aging process can affect the level and efficiency of autophagy [74]. The ability of autophagy to function decreases with age, and this is a bi-directional regulatory process [75]. The mTOR signal is one of the regulatory factors for autophagy, cellular homeostasis and lifespan [76]. Muscle contraction can induce the production of the endogenous peptide apelin, which is a promoter of mitochondrial biogenesis and autophagy. Muscle aging can be combated by targeting muscle stem cells [77].

### 4.1 Age-related skeletal diseases

**Figure 2.**
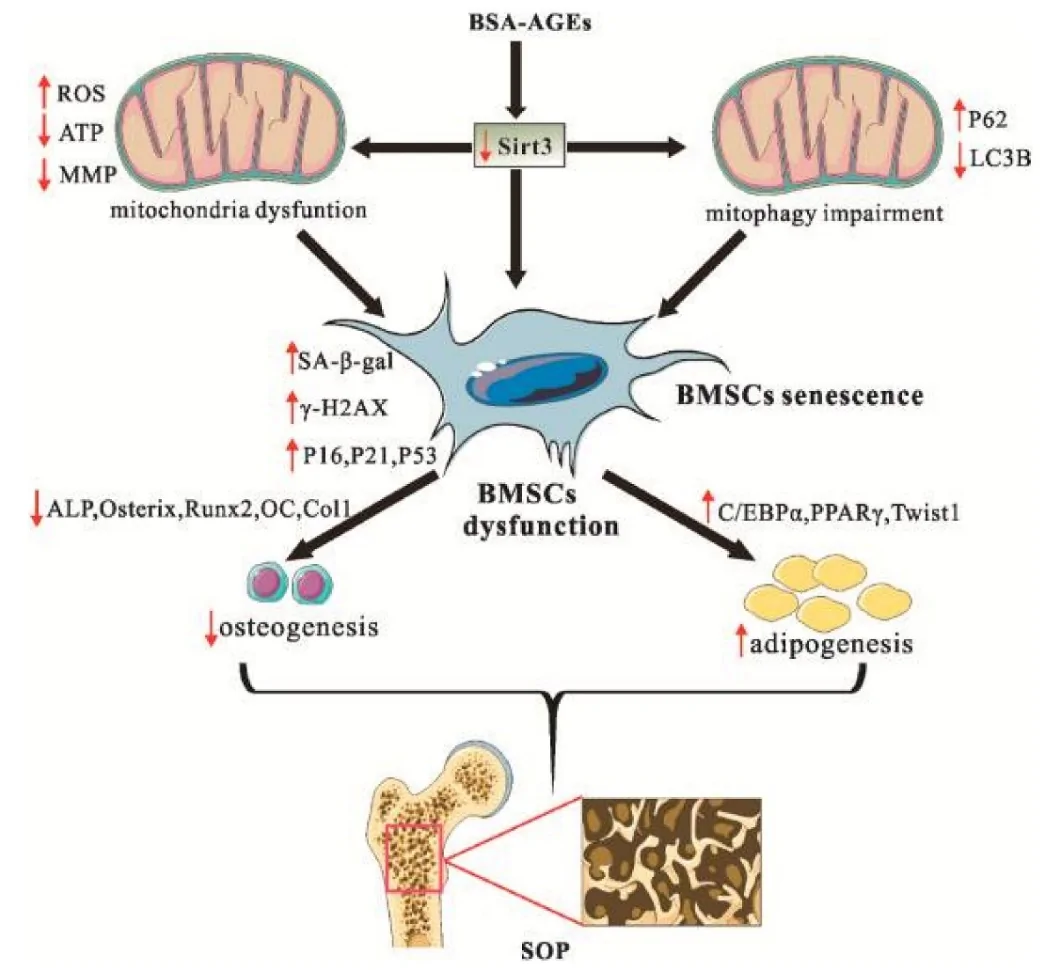
The mechanism of Sirt3-mediated mitophagy regulated AGEs-induced BMSCs senescence and senile osteoporosis [79].

The major age-related skeletal disease is osteoporosis (OP, a condition where bones become thin and lose their strength). In senile osteoporosis, there is a decrease in the activity of autophagy. OPTN is a protein that is involved in selective autophagy, and FABP3 is a novel autophagic substrate of OPTN. Reduced expression of FABP3 results in decreased bone mass, increased adipogenic capacity, decreased osteogenesis and decreased autophagic capacity [78]. The inhibition of mitochondrial autophagy can induce the senescence of bone marrow mesenchymal stem cells, which leads to senile osteoporosis. Aging and the progression of senile osteoporosis can be exacerbated by the deposition of advanced glycation end products. Sirt3 is an enzyme that is a regulator of mitochondrial metabolism and may be an ameliorator of this condition [79] (Figure 2). In the study by Liu et al, it was found that knockdown of LRRc17 activates mitochondrial autophagy by inhibiting the mTOR/PI3K pathway, thereby reducing mitochondrial dysfunction and inhibiting the senescence of bone marrow mesenchymal stem cells by a mechanism similar to that of rapamycin [80]. Rapamycin is an immunosuppressive agent that can regulate the activity of T cells and improve the inflammatory response [81].

### 4.2 Age-related Joint Diseases

**Figure 3.**
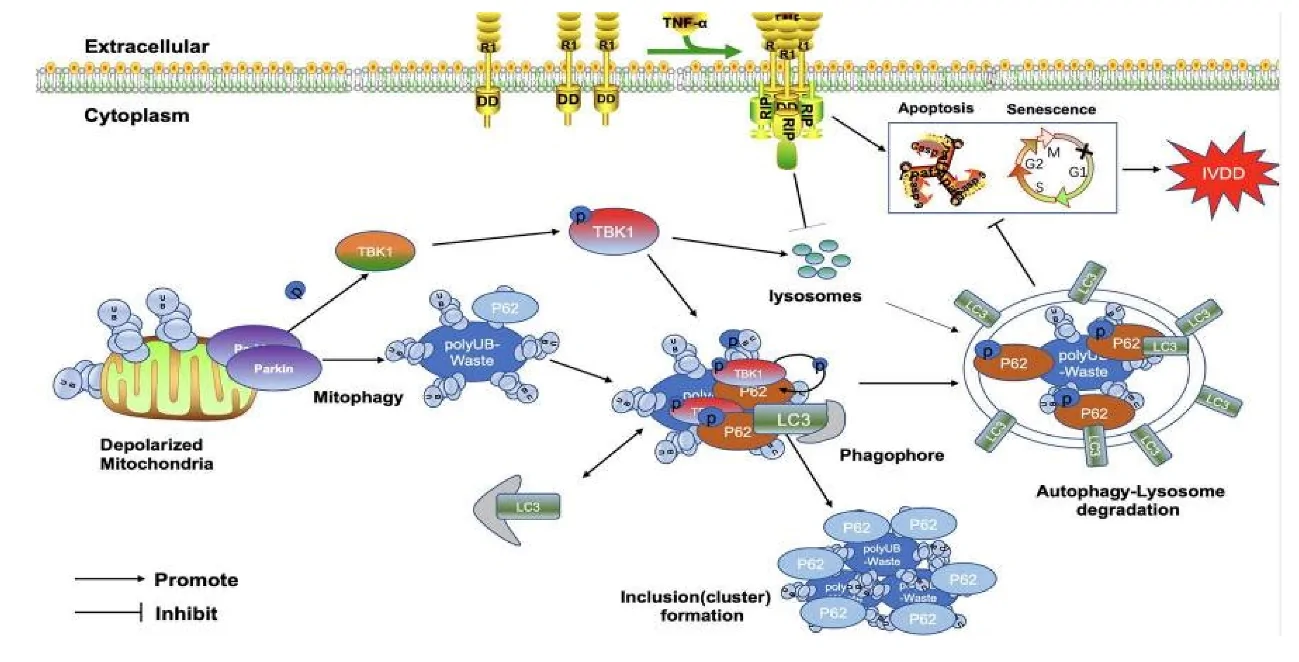
Tank binding kinase 1 (TBK1) in regulating autophagy and this gene protects against intervertebral disc degeneration (IVDD) through affecting autophagy. TBK1 represents a promising avenue for IVDD treatment [87].

Osteoarthritis (OA, defined as joint cartilage and underlying bone injury disease) is an age-related disorder. Increased levels of p62 in cartilage and decreased levels of microtubule-associated protein LC3 and beclin-1 suggest reduced autophagic function and increased cell senescence in tissues from osteoarthritis patients [82]. Inhibition of autophagy is associated with activation of the NLRP3 inflammasome, which has an adverse effect on bone cell function [83]. Fibroblast-like synoviocytes from arthritic patients show impaired autophagy leading to upregulated senescence-associated secretory phenotype. An increase in autophagic flux can suppress the expression of this phenotype and thereby inhibit cell aging and consequent cartilage destruction [84]. IL-1β induces inflammation, mitochondrial dysfunction and chondrocyte degeneration, while Sirt3 in arthritis treatment can ameliorate arthritis severity and joint damage through inhibition of the PI3K/Akt/mTOR pathway [85]. Rapamycin in the treatment of arthritis may potentially be a therapeutic strategy through the upregulation of autophagic flux and the targeting of aging pathways. Clinical therapy requires multiple injections over a short period of time. In the study by Kaamini M et al, it was found that rapamycin loaded into poly (lactic-co-glycolic acid) microspheres can promote the function of cellular autophagy in arthritis patients and thus prevent the aging process [86]. In addition to the effect on the joints, autophagy and aging also play a role in the supporting structures of the joints. For example, TBK1, a protein kinase involved in immunity and autophagy, is decreased with the degeneration of the intervertebral disc, while its overexpression can inhibit the apoptosis and aging of the nucleus pulposus cells. TBK1 primarily enhances autophagy by promoting the fusion of autophagosomes and lysosomes, clearance of damaged mitochondria and protein aggregates to maintain cellular homeostasis. The activation of TBK1 is dependent on Parkin, which is a mediator of mitochondrial autophagy [87] (Figure 3). Mitochondrial DNA depletion factor 2 is also required to promote mitochondrial autophagy in intervertebral disc cells through the PINK1/Parkin pathway in order to protect the cells from oxidative stress [88]. The transcription factor EB is the major regulator of autophagy flux by initiating autophagy-related genes and lysosomal biogenesis. It can also regulate senescence and apoptosis of nucleus pulposus cells in intervertebral disc degeneration [89]. Quercetin can protect the nucleus pulposus from apoptosis and prevent ECM degeneration through the p38 MAPK autophagy pathway [90].

### 4.3 Muscle and Tendon Aging-Related Diseases

Autophagy is a key process in the regulation of muscle mass and function, and muscle aging is one of the causes of muscle atrophy [91]. Studies have shown that oxidative stress can promote muscle atrophy through the autophagy pathway, which may be due to excessive levels of autophagy [92]. Activating defective mitochondria through targeted mitochondrial autophagy can delay muscle atrophy and promote skeletal muscle mitochondrial autophagy [93]. Research has also shown that mitochondrial autophagy may restore skeletal muscle regeneration in aging satellite cells [94]. The ability to form autophagosomes in muscle may be enhanced by exercise training [95]. It has been observed that sedentary elderly individuals have significantly upregulated autophagy genes, whereas the autophagy genes of elderly athletes approach the levels of young athletes. This suggests that autophagy may act as a mechanism against aging and oxidative stress, and exercise may inhibit excessive autophagy, thereby maintaining skeletal muscle homeostasis and metabolism [96]. Exercise training may increase the ability of autophagosome formation in muscle [97]. Intake of essential amino acids after exercise can reduce autophagy in skeletal muscle and increase muscle protein synthesis after exercise [98]. It shows that appropriate autophagy can maintain the homeostasis of the cell environment, but too much autophagy will affect the homeostasis of the environment.

**Figure 4.**
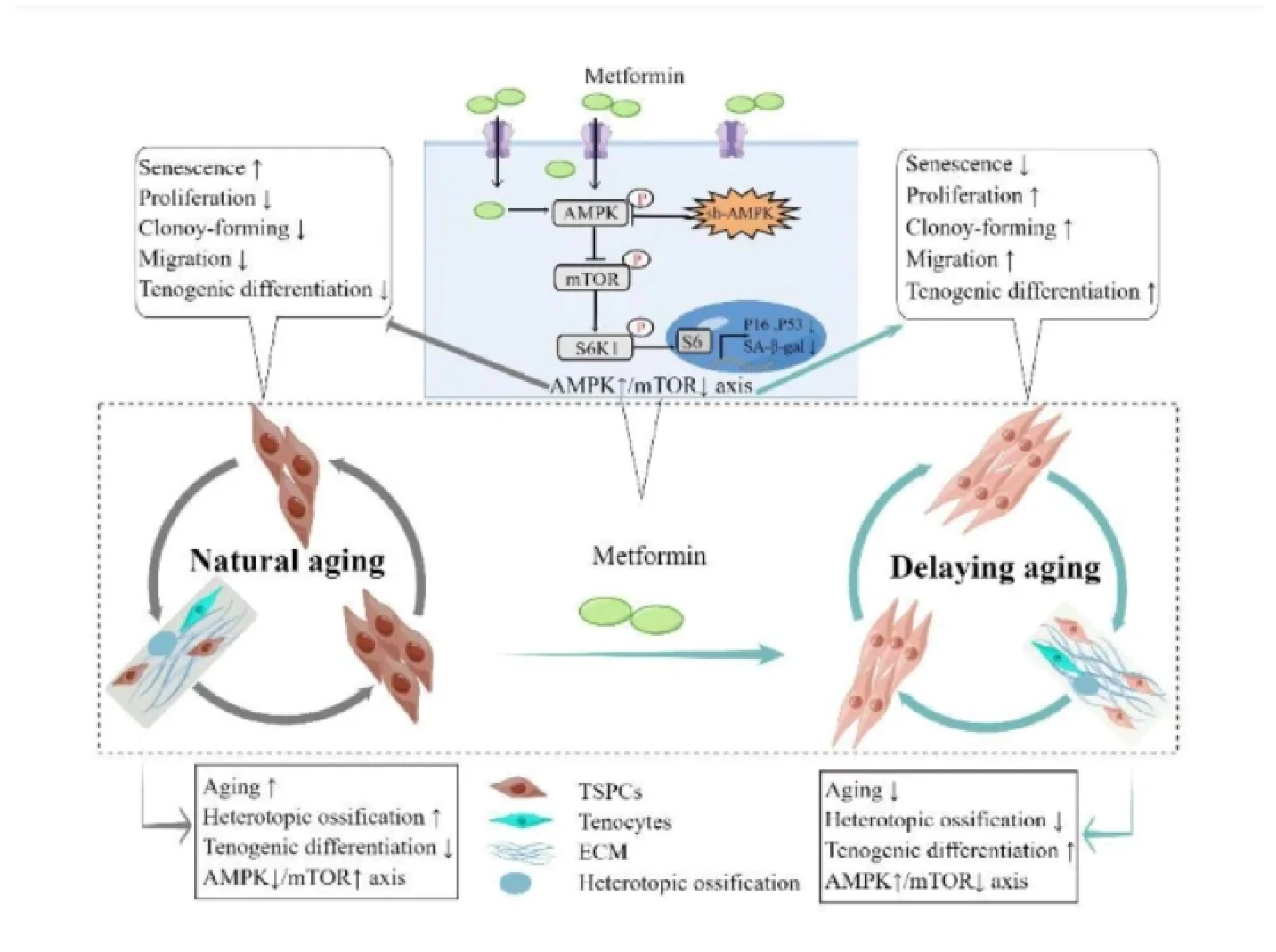
**Age-related tendon disorders are closely linked with tendon stem/progenitor cells (TSPCs) senescence**. This study revealed new insight into TSPCs senescence and proposed a novel therapeutic treatment for age-related tendon disorders by targeting the AMPK/mTOR axis at the early stage of aging [102].

However, there are fewer studies on the diseases caused by autophagy in the aging of muscle and tendon [99]. Autophagy can prevent the loss of self-renewal capacity and stemness induced by oxidative stress in the aging of human tenocytes [100]. Tenocyte senescence occurs with age. Rapamycin has been shown to ameliorate phenotypes associated with senescence, to increase autophagic activity, and to serve as a marker of autophagy [101]. In tendon-derived stem/progenitor cells (TSPCs), the mTOR pathway plays an important role in regulating autophagy, and activation of the AMPK/mTOR axis is associated with aging. Resveratrol may ameliorate the aging of TSPCs by regulating the AMPK/mTOR axis, thereby delaying the aging of tendons and related diseases [102] (Fig. 4).

The decline of various physiological functions is a hallmark of aging. In muscle diseases, aging is often associated with skeletal muscle homeostasis and regeneration. Studies have shown that fibro-adipogenic progenitors (FAPs) represent a cell population that can be therapeutically targeted to improve skeletal health associated with aging, injury and disease [103]. FAPs are a group of muscle progenitor cells that play a critical role in muscle regeneration and maintenance of skeletal muscle fiber size [104]. These pivotal functions of the FAPs are mediated by a complex secretome which acts in a paracrine manner to stimulate muscle satellite cell (SC) division and differentiation. The former are characterized by Pax7 expression and the latter by high PDGFRa expression. Fibrosis, fat infiltration (intramuscular abnormal fat deposition), muscle atrophy and impaired muscle regeneration result from dysregulation of FAPs differentiation [105]. Recent literature reports that these cells are typically quiescent in the absence of muscle injury. Upon injury, cytokines such as IL-4 and/or IL-15 stimulate FAPs proliferation and migration to the site of injury [106]. In particular, activated FAPs secrete signaling factors that instruct SCs to differentiate and proliferate, and in the absence of FAPs, muscle regeneration is compromised. On the other hand, the aging of FAPs is an essential component that leads to the apoptosis of FAPs and impaired muscle regeneration [107]. FAPs do not undergo senescence and are resistant to apoptosis in chronically injured muscles, such as in myopathies. Muscle degeneration therefore occurs when FAPs senescence is inhibited [108-110]. For example, the high levels of fat infiltration and fibrous deposition that are observed in rotator cuff tears have been attributed to FAPs [111-112]. In a mouse model of rotator cuff tears, the levels of FAPs are increased while the apoptosis index is decreased [113]. TGF-β levels increase after injury to the muscle tendon increase the survival rate of FAPs [112-113]. In comparison to other muscle diseases, muscle tendon injuries have a high and concentrated rate of fat infiltration. Shoulder muscles have a greater potential for adipogenesis and have a higher concentration of FAPs than lower limb muscles [114]. Depending on the specific muscle, the amount of FAPs also varies. For example, the percentage of FAPs is higher in the muscles of the shoulder than in the muscles of the quadriceps [115]. These ratios may be of interest in the study of the role of FAPs in various muscle and skeletal disorders [116].

In addition, research has shown that the interaction between FAPs and inflammatory cells is bidirectional. Following muscle injury, immune cells (neutrophils, pro- and anti-inflammatory macrophages, natural killer cells, B cells, and T cells) rapidly accumulate, leading to changes in the proportion of non-immune cells (endothelial cells, smooth muscle cells, glial cells, tendon cells, and fibro-adipogenic progenitor cells) [117]. For example, macrophage polarization has been observed in muscle injury, including rotator cuff atrophy and fat infiltration after large tendon tears. Muscle-resident FAPs fibro/white fat generation leads to fibrosis and fat infiltration, whereas brown/beige fat generation of FAPs promotes shoulder muscle regeneration. This suggests that the transplantation of M2 macrophages may be able to reduce atrophy and fat infiltration in the supraspinatus muscle. However, the intravenous injection of M2 exosomes directly regulates the differentiation of FAPs and significantly reduces the muscle atrophy and fat infiltration in the supraspinatus muscle, thus providing a new therapeutic option for the muscle atrophy and fat infiltration in the rotator cuff [118]. During muscle injury, for example, activated FAPs upregulate the expression of IL-10. This cytokine acts as a central effector in the induction of the transition of macrophage subtypes to their anti-inflammatory phenotype [119]. During muscle regeneration, there is a transition in macrophage phenotype from pro-inflammatory macrophages to anti-inflammatory macrophages. The former secrete cytokines that promote the proliferation of myoblasts, while the latter secrete factors that stimulate the differentiation and fusion of myoblasts. This suggests that FAPs may play a role in switching macrophage phenotypes [120-121]. Mononuclear cells/macrophages also play a critical role in the regulation of FAPs. Changes in cell apoptosis, rather than proliferation, mediate the effect of mononuclear cell/macrophage depletion on FAPs accumulation It has been shown that TNF-α, which is highly secreted by pro-inflammatory macrophages during acute injury, induces FAPs apoptosis [122]. Another pro-inflammatory factor, IL-1α/β, which is secreted by pro-inflammatory macrophages, has been shown to inhibit the adipogenic differentiation of FAPs [123]. There is a transition in macrophage phenotype to anti-inflammatory macrophages secreting higher levels of TGF-β as the regeneration process progresses. This growth factor competes with TNF-α and promotes FAPs survival. TGF-β also down-regulates the expression of the FAPs markers PDGFRα and TCF7L2/TCF4 as well as their downstream signaling pathways [124-125]. TGF-β influences the fate determination of FAPs through pathways such as increasing the survival of FAPs, promoting their proliferation, inhibiting their adipogenic differentiation, and promoting their differentiation into myofibroblasts [113, 126].

**Figure 5.**
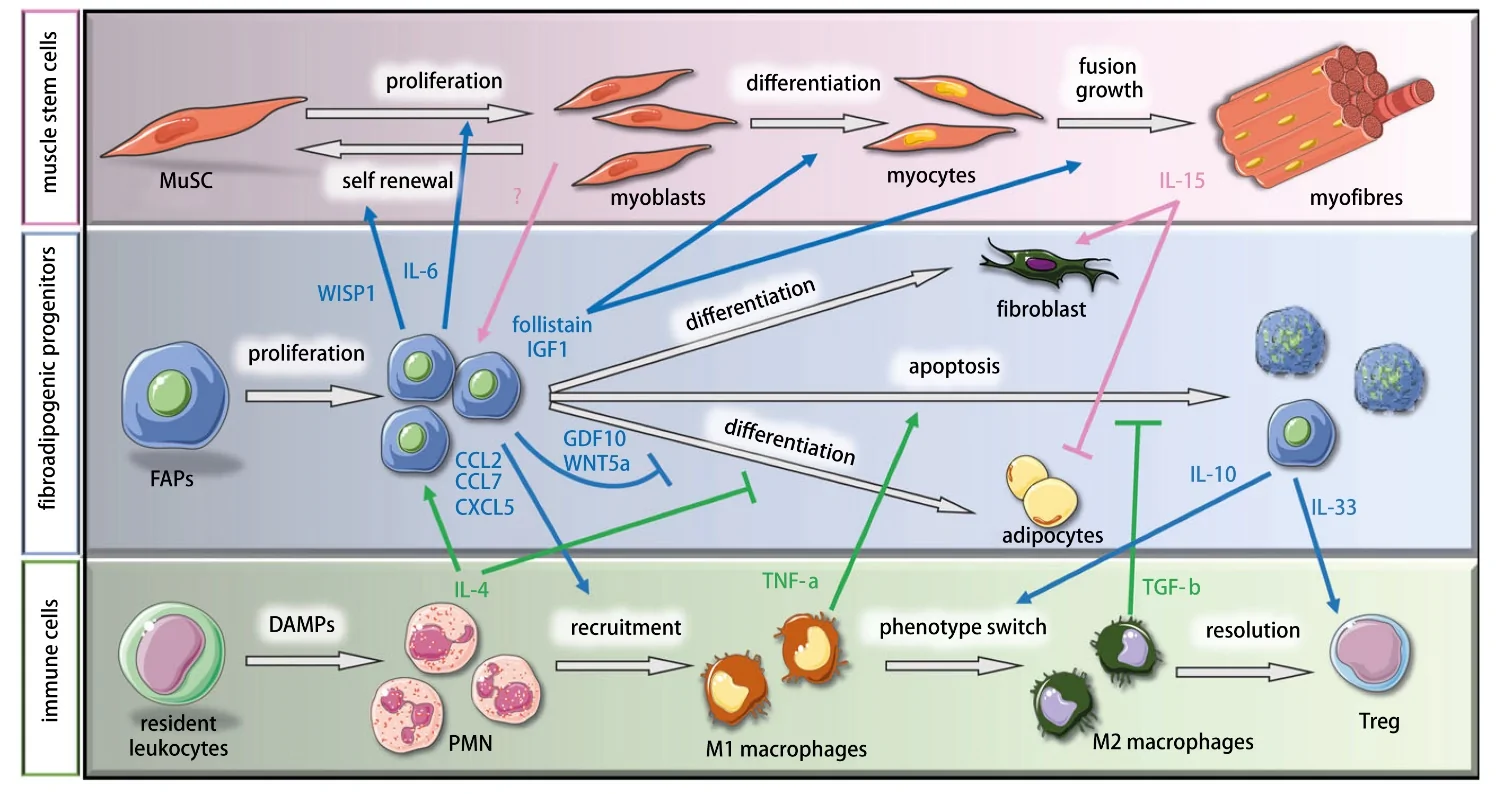
the interaction of FAPs with MuSC and immune cells [127].

New technologies, such as single-cell RNA sequencing, have revealed the cellular heterogeneity of FAPs and the complex regulatory network that regulates them during muscle regeneration. Due to their central role in skeletal muscle pathophysiology, the regulatory mechanisms of FAPs and their cellular and molecular crosstalk with muscle stem cells (MuSCs) have been extensively studied [127] (Figure 5). Recently, the cellular heterogeneity of FAPs and their complex molecular interactions during different stages of muscle regeneration and muscle diseases have been identified by breakthroughs in single-cell sequencing technology. These new insights will play a critical role in the development of novel therapeutic approaches targeting FAPs to limit their accumulation and/or restore their function, thereby reducing fibrofatty deposition and promoting muscle regeneration in muscle diseases.

## 5. Conclusions

The onset of various diseases is often associated with the aging process in the human body. Cellular autophagy provides functions such as anti-inflammation, stress resistance, mitochondrial regulation and protein degradation, and has the ability to facilitate self-renewal in aging cells. It is a molecular process necessary for the maintenance of cellular homeostasis and may have a regulatory role in the delay of aging and age-related diseases. However, the capacity of autophagy is reduced with age. Autophagy and aging exist in a dynamic equilibrium. Appropriate levels of autophagy can ameliorate aging and age-related diseases, whereas excessive levels of autophagy can disrupt the internal environment of the organism. This phenomenon is not only evident in diseases associated with aging in general, but it also plays a role in diseases associated with the musculoskeletal system. Studies have shown that many drugs increase autophagy to regulate aging, therefore, novel approaches for the treatment of aging and related diseases may be developed by exploring the molecular mechanisms between autophagy and aging using autophagy-targeted activators or inhibitors for early prevention and intervention of skeletal muscle aging. Obviously, since exercise and glucose and lipid metabolism play a critical role in homeostatic regulation, attention to this field can help improve exercise function and accelerate "healthy aging" in older people.

Removing senescent cells may prevent neighboring healthy cells from entering the vicious cycle of senescence. How to promote repair of existing tissue damage is another important issue that needs to be addressed. In the future, in addition to in-depth studies of the regulatory effects of cells of different origins on the microenvironment of the motor system and the crosstalk mechanisms with different senescent cells, combined with new gene sequencing and bioinformatics analysis technologies, such as single-cell nuclear transcriptome and single-cell nuclear chromatin openome, can be selected to construct a multimodal cell atlas of the aging human skeletal muscle to help further explore the molecular mechanisms of senescence, which may provide new ideas for cellular rejuvenation and in situ repair of tissue damage.

## Author Contributions

H.Z. and W.G. proposed the research idea and wrote the original manuscript; H.Z. was responsible for drawing and revising the manuscript; G.W. and Y.Y was responsible for the selection of the topic and revision of the manuscript, and management of resources and project. All authors have read and agreed to the published version of the manuscript.

## Data Availability

No data were created for this review article. All data analyzed were included in the manuscript. All data or information can be provided on demand by the corresponding author.

## References

[1] Ogrodnik M, Miwa S, Tchkonia T, Tiniakos D, Wilson CL, Lahat A, et al. (2017). Cellular senescence drives age-dependent hepatic steatosis. Nat Commun, 8:15691. 10.1038/ncomms15691 PMC5474745 28608850

[2] Dillin A, Gottschling DE, Nyström T (2014). The good and the bad of being connected: the integrons of aging. Curr Opin Cell Biol, 26:107-112. 10.1016/j.ceb.2013.12.003 PMC3927154 24529252

[3] de Farias JO, Sousa M, Martins DCM, Oliveira MA, Takahashi I, Sousa LB, et al. (2024). Senescence on dental pulp cells: effects on morphology, migration, proliferation, and immune response. J Endod. 10.1016/j.joen.2023.12.009 38211820

[4] Hayflick L, Moorhead PS (1961). The serial cultivation of human diploid cell strains. Exp Cell Res, 25:585-621. 10.1016/0014-4827(61)90192-6 13905658

[5] Gorgoulis V, Adams PD, Alimonti A, Bennett DC, Bischof O, Bishop C, et al. (2019). Cellular Senescence: Defining a Path Forward. Cell, 179:813-827. 10.1016/j.cell.2019.10.005 31675495

[6] Campisi J (2013). Aging, cellular senescence, and cancer. Annu Rev Physiol, 75:685-705. 10.1146/annurev-physiol-030212-183653 PMC4166529 23140366

[7] Davaapil H, Brockes JP, Yun MH (2017). Conserved and novel functions of programmed cellular senescence during vertebrate development. Development, 144:106-114. 10.1242/dev.138222 PMC5278627 27888193

[8] Calcinotto A, Kohli J, Zagato E, Pellegrini L, Demaria M, Alimonti A (2019). Cellular Senescence: Aging, Cancer, and Injury. Physiol Rev, 99:1047-1078. 10.1152/physrev.00020.2018 30648461

[9] Taketani H, Nishikawa T, Nakajima H, Kodo K, Sugimoto S, Aoi W, et al. (2019). Aging-associated impairment in metabolic compensation by subcutaneous adipose tissue promotes diet-induced fatty liver disease in mice. Diabetes Metab Syndr Obes, 12:1473-1492. 10.2147/DMSO.S214093 PMC6711723 31692556

[10] Kim IH, Kisseleva T, Brenner DA (2015). Aging and liver disease. Curr Opin Gastroenterol, 31:184-191. 10.1097/MOG.0000000000000176 PMC4736713 25850346

[11] Muñoz-Espín D, Serrano M (2014). Cellular senescence: from physiology to pathology. Nat Rev Mol Cell Biol, 15:482-496. 10.1038/nrm3823 24954210

[12] Childs BG, Durik M, Baker DJ, van Deursen JM (2015). Cellular senescence in aging and age-related disease: from mechanisms to therapy. Nat Med, 21:1424-1435. 10.1038/nm.4000 PMC4748967 26646499

[13] Yoshida H (2007). ER stress and diseases. Febs j, 274:630-658. 10.1111/j.1742-4658.2007.05639.x 17288551

[14] Hernandez-Segura A, Nehme J, Demaria M (2018). Hallmarks of Cellular Senescence. Trends Cell Biol, 28:436-453. 10.1016/j.tcb.2018.02.001 29477613

[15] Wu CJ, Liu RX, Huan SW, Tang W, Zeng YK, Zhang JC, et al. (2022). Senescent skeletal cells cross-talk with synovial cells plays a key role in the pathogenesis of osteoarthritis. Arthritis Res Ther, 24:59. 10.1186/s13075-022-02747-4 PMC8883702 35227288

[16] Dodig S, Čepelak I, Pavić I (2019). Hallmarks of senescence and aging. Biochem Med (Zagreb), 29:030501. 10.11613/BM.2019.030501 PMC6610675 31379458

[17] Greene MA, Loeser RF (2015). Aging-related inflammation in osteoarthritis. Osteoarthritis Cartilage, 23:1966-1971. 10.1016/j.joca.2015.01.008 PMC4630808 26521742

[18] Li Y, Zhao H, Huang X, Tang J, Zhang S, Li Y, et al. (2018). Embryonic senescent cells re-enter cell cycle and contribute to tissues after birth. Cell Res, 28:775-778. 10.1038/s41422-018-0050-6 PMC6028486 29872106

[19] Storer M, Mas A, Robert-Moreno A, Pecoraro M, Ortells MC, Di Giacomo V, et al. (2013). Senescence is a developmental mechanism that contributes to embryonic growth and patterning. Cell, 155:1119-1130. 10.1016/j.cell.2013.10.041 24238961

[20] Demaria M, Ohtani N, Youssef SA, Rodier F, Toussaint W, Mitchell JR, et al. (2014). An essential role for senescent cells in optimal wound healing through secretion of PDGF-AA. Dev Cell, 31:722-733. 10.1016/j.devcel.2014.11.012 PMC4349629 25499914

[21] Helman A, Klochendler A, Azazmeh N, Gabai Y, Horwitz E, Anzi S, et al. (2016). p16(Ink4a)-induced senescence of pancreatic beta cells enhances insulin secretion. Nat Med, 22:412-420. 10.1038/nm.4054 PMC5546206 26950362

[22] He S, Sharpless NE (2017). Senescence in Health and Disease. Cell, 169:1000-1011. 10.1016/j.cell.2017.05.015 PMC5643029 28575665

[23] Gonzales MM, Garbarino VR, Marques Zilli E, Petersen RC, Kirkland JL, Tchkonia T, et al. (2022). Senolytic Therapy to Modulate the Progression of Alzheimer's Disease (SToMP-AD): A Pilot Clinical Trial. J Prev Alzheimers Dis, 9:22-29. 10.14283/jpad.2021.62 PMC8612719 35098970

[24] Li Z, Cai K, Sun Y, Zhou D, Yan J, Luo S, et al. (2023). Folic acid protects against age-associated apoptosis and telomere attrition of neural stem cells in senescence-accelerated mouse prone 8. Appl Physiol Nutr Metab, 48:393-402. 10.1139/apnm-2022-0111 36809211

[25] Mandraffino G, Aragona CO, Basile G, Cairo V, Mamone F, Morace C, et al. (2017). CD34+ cell count predicts long lasting life in the oldest old. Mech Ageing Dev, 164:139-145. 10.1016/j.mad.2017.03.003 28322848

[26] Yaskolka Meir A, Keller M, Bernhart SH, Rinott E, Tsaban G, Zelicha H, et al. (2021). Lifestyle weight-loss intervention may attenuate methylation aging: the CENTRAL MRI randomized controlled trial. Clin Epigenetics, 13:48. 10.1186/s13148-021-01038-0 PMC7934393 33663610

[27] Mannick JB, Del Giudice G, Lattanzi M, Valiante NM, Praestgaard J, Huang B, et al. (2014). mTOR inhibition improves immune function in the elderly. Sci Transl Med, 6:268ra179. 10.1126/scitranslmed.3009892 25540326

[28] Chatsirisupachai K, Palmer D, Ferreira S, de Magalhães JP (2019). A human tissue-specific transcriptomic analysis reveals a complex relationship between aging, cancer, and cellular senescence. Aging Cell, 18:e13041. 10.1111/acel.13041 PMC6826163 31560156

[29] Li CW, Yu K, Shyh-Chang N, Li GX, Jiang LJ, Yu SL, et al. (2019). Circulating factors associated with sarcopenia during ageing and after intensive lifestyle intervention. J Cachexia Sarcopenia Muscle, 10:586-600. 10.1002/jcsm.12417 PMC6596393 30969486

[30] Zhang Y, Liu L, Qi Y, Lou J, Chen Y, Liu C, et al. (2024). Lactic acid promotes nucleus pulposus cell senescence and corresponding intervertebral disc degeneration via interacting with Akt. Cell Mol Life Sci, 81:24. 10.1007/s00018-023-05094-y PMC11071984 38212432

[31] Chen Y, Lin J, Chen J, Huang C, Zhang Z, Wang J, et al. (2020). Mfn2 is involved in intervertebral disc degeneration through autophagy modulation. Osteoarthritis Cartilage, 28:363-374. 10.1016/j.joca.2019.12.009 31926268

[32] Jia S, Yu Z, Bai L (2023). Exerkines and osteoarthritis. Front Physiol, 14:1302769. 10.3389/fphys.2023.1302769 PMC10722202 38107476

[33] Xu M, Tchkonia T, Ding H, Ogrodnik M, Lubbers ER, Pirtskhalava T, et al. (2015). JAK inhibition alleviates the cellular senescence-associated secretory phenotype and frailty in old age. Proc Natl Acad Sci U S A, 112:E6301-6310. 10.1073/pnas.1515386112 PMC4655580 26578790

[34] Feng Y, He D, Yao Z, Klionsky DJ (2014). The machinery of macroautophagy. Cell Res, 24:24-41. 10.1038/cr.2013.168 PMC3879710 24366339

[35] Song H, Zhu Y, Hu C, Liu Q, Jin Y, Tang P, et al. (2024). Selective Autophagy Receptor NBR1 Retards Nucleus Pulposus Cell Senescence by Directing the Clearance of SRBD1. Int J Biol Sci, 20:701-717. 10.7150/ijbs.90186 PMC10758090 38169523

[36] Liu S, Yao S, Yang H, Liu S, Wang Y (2023). Autophagy: Regulator of cell death. Cell Death Dis, 14:648. 10.1038/s41419-023-06154-8 PMC10551038 37794028

[37] Wang Y, Tang M (2020). PM2.5 induces autophagy and apoptosis through endoplasmic reticulum stress in human endothelial cells. Sci Total Environ, 710:136397. 10.1016/j.scitotenv.2019.136397 32050373

[38] Zois CE, Koukourakis MI (2009). Radiation-induced autophagy in normal and cancer cells: towards novel cytoprotection and radio-sensitization policies? Autophagy, 5:442-450. 10.4161/auto.5.4.7667 19164950

[39] Shaikh S, Ahmad K, Ahmad SS, Lee EJ, Lim JH, Beg MMA, et al. (2021). Natural Products in Therapeutic Management of Multineurodegenerative Disorders by Targeting Autophagy. Oxid Med Cell Longev, 2021:6347792. 10.1155/2021/6347792 PMC8455192 34557265

[40] Wilhelm LP, Zapata-Muñoz J, Villarejo-Zori B, Pellegrin S, Freire CM, Toye AM, et al. (2022). BNIP3L/NIX regulates both mitophagy and pexophagy. Embo j, 41:e111115. 10.15252/embj.2022111115 PMC9753467 36215693

[41] Liu J, Liu Y, Wang Y, Li C, Xie Y, Klionsky DJ, et al. (2023). TMEM164 is a new determinant of autophagy-dependent ferroptosis. Autophagy, 19:945-956. 10.1080/15548627.2022.2111635 PMC9980451 35947500

[42] Torii S, Yoshida T, Arakawa S, Honda S, Nakanishi A, Shimizu S (2016). Identification of PPM1D as an essential Ulk1 phosphatase for genotoxic stress-induced autophagy. EMBO Rep, 17:1552-1564. 10.15252/embr.201642565 PMC5090708 27670885

[43] Gluschko A, Farid A, Herb M, Grumme D, Krönke M, Schramm M (2022). Macrophages target Listeria monocytogenes by two discrete non-canonical autophagy pathways. Autophagy, 18:1090-1107. 10.1080/15548627.2021.1969765 PMC9196813 34482812

[44] Rigo A, Vinante F (2016). The antineoplastic agent α-bisabolol promotes cell death by inducing pores in mitochondria and lysosomes. Apoptosis, 21:917-927. 10.1007/s10495-016-1257-y 27278818

[45] Shen Y, Malik SA, Amir M, Kumar P, Cingolani F, Wen J, et al. (2020). Decreased Hepatocyte Autophagy Leads to Synergistic IL-1β and TNF Mouse Liver Injury and Inflammation. Hepatology, 72:595-608. 10.1002/hep.31209 PMC8114460 32108953

[46] Dai W, Wang M, Wang P, Wen J, Wang J, Cha S, et al. (2021). lncRNA NEAT1 ameliorates LPS-induced inflammation in MG63 cells by activating autophagy and suppressing the NLRP3 inflammasome. Int J Mol Med, 47:607-620. 10.3892/ijmm.2020.4827 PMC7797466 33416115

[47] Bharath LP, Agrawal M, McCambridge G, Nicholas DA, Hasturk H, Liu J, et al. (2020). Metformin Enhances Autophagy and Normalizes Mitochondrial Function to Alleviate Aging-Associated Inflammation. Cell Metab, 32:44-55.e46. 10.1016/j.cmet.2020.04.015 PMC7217133 32402267

[48] Luo J, Wang J, Zhang J, Sang A, Ye X, Cheng Z, et al. (2022). Nrf2 Deficiency Exacerbated CLP-Induced Pulmonary Injury and Inflammation through Autophagy- and NF-κB/PPARγ-Mediated Macrophage Polarization. Cells, 11. 10.3390/cells11233927 PMC9735993 36497185

[49] Gao K, Zong H, Hou K, Zhang Y, Zhang R, Zhao D, et al. (2022). p53N236S Activates Autophagy in Response to Hypoxic Stress Induced by DFO. Genes(Basel), 13. 10.3390/genes13050763 PMC9141750 35627147

[50] Wu Z, Wang H, Fang S, Xu C (2018). Roles of endoplasmic reticulum stress and autophagy on H2O2-induced oxidative stress injury in HepG2 cells. Mol Med Rep, 18:4163-4174. 10.3892/mmr.2018.9443 PMC6172379 30221706

[51] Shao Z, Ni L, Hu S, Xu T, Meftah Z, Yu Z, et al. (2021). RNA-binding protein HuR suppresses senescence through Atg7 mediated autophagy activation in diabetic intervertebral disc degeneration. Cell Prolif, 54:e12975. 10.1111/cpr.12975 PMC7848958 33372336

[52] Wang Y, Wang M, Liu Y, Tao H, Banerjee S, Srinivasan S, et al. (2022). Integrated regulation of stress responses, autophagy and survival by altered intracellular iron stores. Redox Biol, 55:102407. 10.1016/j.redox.2022.102407 PMC9294649 35853304

[53] Ha J, Park SB (2021). Callyspongiolide kills cells by inducing mitochondrial dysfunction via cellular iron depletion. Commun Biol, 4:1123. 10.1038/s42003-021-02643-8 PMC8460830 34556786

[54] Saha B, Olsvik H, Williams GL, Oh S, Evjen G, Sjøttem E, et al. (2023). TBK1 is ubiquitinated by TRIM5α to assemble mitophagy machinery. bioRxiv. 10.1016/j.celrep.2024.114294 PMC11216866 38814780

[55] Guan R, Yuan L, Li J, Wang J, Li Z, Cai Z, et al. (2022). Bone morphogenetic protein 4 inhibits pulmonary fibrosis by modulating cellular senescence and mitophagy in lung fibroblasts. Eur Respir J, 60. 10.1183/13993003.02307-2021 PMC9808813 35777761

[56] Li J, Wu X, He Y, Wu S, Guo E, Feng Y, et al. (2021). PINK1 antagonize intracerebral hemorrhage by promoting mitochondrial autophagy. Ann Clin Transl Neurol, 8:1951-1960. 10.1002/acn3.51425 PMC8528457 34453779

[57] Xiang H, Zhou M, Li Y, Zhou L, Wang R (2023). Drug discovery by targeting the protein-protein interactions involved in autophagy. Acta Pharm Sin B, 13:4373-4390. 10.1016/j.apsb.2023.07.016 PMC10638514 37969735

[58] Shpilka T, Weidberg H, Pietrokovski S, Elazar Z (2011). Atg8: an autophagy-related ubiquitin-like protein family. Genome Biol, 12:226. 10.1186/gb-2011-12-7-226 PMC3218822 21867568

[59] Behrends C, Sowa ME, Gygi SP, Harper JW (2010). Network organization of the human autophagy system. Nature, 466:68-76. 10.1038/nature09204 PMC2901998 20562859

[60] Ma X, Lu C, Chen Y, Li S, Ma N, Tao X, et al. (2022). CCT2 is an aggrephagy receptor for clearance of solid protein aggregates. Cell, 185:1325-1345.e1322. 10.1016/j.cell.2022.03.005 35366418

[61] Hentilä J, Hulmi JJ, Laakkonen EK, Ahtiainen JP, Suominen H, Korhonen MT (2020). Sprint and Strength Training Modulates Autophagy and Proteostasis in Aging Sprinters. Med Sci Sports Exerc, 52:1948-1959. 10.1249/MSS.0000000000002340 32205677

[62] Tchkonia T, Kirkland JL (2018). Aging, Cell Senescence, and Chronic Disease: Emerging Therapeutic Strategies. Jama, 320:1319-1320. 10.1001/jama.2018.12440 30242336

[63] Herranz N, Gil J (2018). Mechanisms and functions of cellular senescence. J Clin Invest, 128:1238-1246. 10.1172/JCI95148 PMC5873888 29608137

[64] Birch J, Barnes PJ, Passos JF (2018). Mitochondria, telomeres and cell senescence: Implications for lung ageing and disease. Pharmacol Ther, 183:34-49. 10.1016/j.pharmthera.2017.10.005 28987319

[65] Madeo F, Tavernarakis N, Kroemer G (2010). Can autophagy promote longevity? Nat Cell Biol, 12:842-846. 10.1038/ncb0910-842 20811357

[66] Yu L, Chen Y, Tooze SA (2018). Autophagy pathway: Cellular and molecular mechanisms. Autophagy, 14:207-215. 10.1080/15548627.2017.1378838 PMC5902171 28933638

[67] Yuen I (1992). A student from Hong Kong looks at American health care costs. Imprint, 39:76-80. 1295836

[68] Pickford F, Masliah E, Britschgi M, Lucin K, Narasimhan R, Jaeger PA, et al. (2008). The autophagy-related protein beclin 1 shows reduced expression in early Alzheimer disease and regulates amyloid beta accumulation in mice. J Clin Invest, 118:2190-2199. 10.1172/JCI33585 PMC2391284 18497889

[69] Kesireddy VS, Chillappagari S, Ahuja S, Knudsen L, Henneke I, Graumann J, et al. (2019). Susceptibility of microtubule-associated protein 1 light chain 3β (MAP1LC3B/LC3B) knockout mice to lung injury and fibrosis. Faseb j, 33:12392-12408. 10.1096/fj.201900854R PMC6902724 31431059

[70] Araya J, Kojima J, Takasaka N, Ito S, Fujii S, Hara H, et al. (2013). Insufficient autophagy in idiopathic pulmonary fibrosis. Am J Physiol Lung Cell Mol Physiol, 304:L56-69. 10.1152/ajplung.00213.2012 23087019

[71] Ricci A, Cherubini E, Scozzi D, Pietrangeli V, Tabbì L, Raffa S, et al. (2013). Decreased expression of autophagic beclin 1 protein in idiopathic pulmonary fibrosis fibroblasts. J Cell Physiol, 228:1516-1524. 10.1002/jcp.24307 23444126

[72] Rangarajan S, Kurundkar A, Kurundkar D, Bernard K, Sanders YY, Ding Q, et al. (2016). Novel Mechanisms for the Antifibrotic Action of Nintedanib. Am J Respir Cell Mol Biol, 54:51-59. 10.1165/rcmb.2014-0445OC PMC4742925 26072676

[73] Youle RJ, Narendra DP (2011). Mechanisms of mitophagy. Nat Rev Mol Cell Biol, 12:9-14. 10.1038/nrm3028 PMC4780047 21179058

[74] Robinson MM, Dasari S, Konopka AR, Johnson ML, Manjunatha S, Esponda RR, et al. (2017). Enhanced Protein Translation Underlies Improved Metabolic and Physical Adaptations to Different Exercise Training Modes in Young and Old Humans. Cell Metab, 25:581-592. 10.1016/j.cmet.2017.02.009 PMC5423095 28273480

[75] Chen ML, Hong CG, Yue T, Li HM, Duan R, Hu WB, et al. (2021). Inhibition of miR-331-3p and miR-9-5p ameliorates Alzheimer's disease by enhancing autophagy. Theranostics, 11:2395-2409. 10.7150/thno.47408 PMC7797673 33500732

[76] Chang K, Kang P, Liu Y, Huang K, Miao T, Sagona AP, et al. (2020). TGFB-INHB/activin signaling regulates age-dependent autophagy and cardiac health through inhibition of MTORC2. Autophagy, 16:1807-1822. 10.1080/15548627.2019.1704117 PMC8386626 31884871

[77] Vinel C, Lukjanenko L, Batut A, Deleruyelle S, Pradère JP, Le Gonidec S, et al. (2018). The exerkine apelin reverses age-associated sarcopenia. Nat Med, 24:1360-1371. 10.1038/s41591-018-0131-6 30061698

[78] Liu ZZ, Hong CG, Hu WB, Chen ML, Duan R, Li HM, et al. (2021). Autophagy receptor OPTN (optineurin) regulates mesenchymal stem cell fate and bone-fat balance during aging by clearing FABP3. Autophagy, 17:2766-2782. 10.1080/15548627.2020.1839286 PMC8526045 33143524

[79] Guo Y, Jia X, Cui Y, Song Y, Wang S, Geng Y, et al. (2021). Sirt3-mediated mitophagy regulates AGEs-induced BMSCs senescence and senile osteoporosis. Redox Biol, 41:101915. 10.1016/j.redox.2021.101915 PMC7930642 33662874

[80] Liu F, Yuan Y, Bai L, Yuan L, Li L, Liu J, et al. (2021). LRRc17 controls BMSC senescence via mitophagy and inhibits the therapeutic effect of BMSCs on ovariectomy-induced bone loss. Redox Biol, 43:101963. 10.1016/j.redox.2021.101963 PMC8066428 33865167

[81] Bagherpour B, Salehi M, Jafari R, Bagheri A, Kiani-Esfahani A, Edalati M, et al. (2018). Promising effect of rapamycin on multiple sclerosis. Mult Scler Relat Disord, 26:40-45. 10.1016/j.msard.2018.08.009 30219744

[82] Gu Y, Yan R, Wang Y, Zeng Y, Yao Q (2022). High TRB3 expression induces chondrocyte autophagy and senescence in osteoarthritis cartilage. Aging (Albany NY), 14:5366-5375. 10.18632/aging.204066 PMC9320551 35776529

[83] Liao S, Zheng Q, Shen H, Yang G, Xu Y, Zhang X, et al. (2023). HECTD1-Mediated Ubiquitination and Degradation of Rubicon Regulates Autophagy and Osteoarthritis Pathogenesis. Arthritis Rheumatol, 75:387-400. 10.1002/art.42369 36121967

[84] Chen X, Gong W, Shao X, Shi T, Zhang L, Dong J, et al. (2022). METTL3-mediated m(6)A modification of ATG7 regulates autophagy-GATA4 axis to promote cellular senescence and osteoarthritis progression. Ann Rheum Dis, 81:87-99. 10.1136/annrheumdis-2021-221091 34706873

[85] Xu K, He Y, Moqbel SAA, Zhou X, Wu L, Bao J (2021). SIRT3 ameliorates osteoarthritis via regulating chondrocyte autophagy and apoptosis through the PI3K/Akt/mTOR pathway. Int J Biol Macromol, 175:351-360. 10.1016/j.ijbiomac.2021.02.029 33556400

[86] Dhanabalan KM, Dravid AA, Agarwal S, Sharath RK, Padmanabhan AK, Agarwal R (2023). Intra-articular injection of rapamycin microparticles prevent senescence and effectively treat osteoarthritis. Bioeng Transl Med, 8:e10298. 10.1002/btm2.10298 PMC9842044 36684078

[87] Hu S, Chen L, Al Mamun A, Ni L, Gao W, Lin Y, et al. (2021). The therapeutic effect of TBK1 in intervertebral disc degeneration via coordinating selective autophagy and autophagic functions. J Adv Res, 30:1-13. 10.1016/j.jare.2020.08.011 PMC8132185 34026282

[88] Liu Y, Yao C, Sheng B, Zhi S, Chen X, Ding P, et al. (2023). Inhibition of USP30 Promotes Mitophagy by Regulating Ubiquitination of MFN2 by Parkin to Attenuate Early Brain Injury After SAH. Transl Stroke Res. 10.1007/s12975-023-01228-3 PMC11976779 38147294

[89] Zheng G, Pan Z, Zhan Y, Tang Q, Zheng F, Zhou Y, et al. (2019). TFEB protects nucleus pulposus cells against apoptosis and senescence via restoring autophagic flux. Osteoarthritis Cartilage, 27:347-357. 10.1016/j.joca.2018.10.011 30414849

[90] Zhang S, Liang W, Abulizi Y, Xu T, Cao R, Xun C, et al. (2021). Quercetin Alleviates Intervertebral Disc Degeneration by Modulating p38 MAPK-Mediated Autophagy. Biomed Res Int, 2021:6631562. 10.1155/2021/6631562 PMC8133869 34055990

[91] Masiero E, Agatea L, Mammucari C, Blaauw B, Loro E, Komatsu M, et al. (2009). Autophagy is required to maintain muscle mass. Cell Metab, 10:507-515. 10.1016/j.cmet.2009.10.008 19945408

[92] Leduc-Gaudet JP, Franco-Romero A, Cefis M, Moamer A, Broering FE, Milan G, et al. (2023). MYTHO is a novel regulator of skeletal muscle autophagy and integrity. Nat Commun, 14:1199. 10.1038/s41467-023-36817-1 PMC9981687 36864049

[93] Singh A, D'Amico D, Andreux PA, Fouassier AM, Blanco-Bose W, Evans M, et al. (2022). Urolithin A improves muscle strength, exercise performance, and biomarkers of mitochondrial health in a randomized trial in middle-aged adults. Cell Rep Med, 3:100633. 10.1016/j.xcrm.2022.100633 PMC9133463 35584623

[94] Hong X, Isern J, Campanario S, Perdiguero E, Ramírez-Pardo I, Segalés J, et al. (2022). Mitochondrial dynamics maintain muscle stem cell regenerative competence throughout adult life by regulating metabolism and mitophagy. Cell Stem Cell, 29:1298-1314.e1210. 10.1016/j.stem.2022.07.009 35998641

[95] Fritzen AM, Madsen AB, Kleinert M, Treebak JT, Lundsgaard AM, Jensen TE, et al. (2016). Regulation of autophagy in human skeletal muscle: effects of exercise, exercise training and insulin stimulation. J Physiol, 594:745-761. 10.1113/JP271405 PMC5341711 26614120

[96] Zampieri S, Pietrangelo L, Loefler S, Fruhmann H, Vogelauer M, Burggraf S, et al. (2015). Lifelong physical exercise delays age-associated skeletal muscle decline. J Gerontol A Biol Sci Med Sci, 70:163-173. 10.1093/gerona/glu006 24550352

[97] Møller AB, Voss TS, Vendelbo MH, Pedersen SB, Møller N, Jessen N (2018). Insulin inhibits autophagy signaling independent of counterregulatory hormone levels but does not affect the effects of exercise. J Appl Physiol (1985), 125:1204-1209. 10.1152/japplphysiol.00490.2018 30070610

[98] Dickinson JM, Reidy PT, Gundermann DM, Borack MS, Walker DK, D'Lugos AC, et al. (2017). The impact of postexercise essential amino acid ingestion on the ubiquitin proteasome and autophagosomal-lysosomal systems in skeletal muscle of older men. J Appl Physiol (1985), 122:620-630. 10.1152/japplphysiol.00632.2016 PMC5401961 27586837

[99] Montagna C, Svensson RB, Bayer ML, Rizza S, Maiani E, Yeung CC, et al. (2022). Autophagy guards tendon homeostasis. Cell Death Dis, 13:402. 10.1038/s41419-022-04824-7 PMC9035152 35461310

[100] Chen H, Ge HA, Wu GB, Cheng B, Lu Y, Jiang C (2016). Autophagy Prevents Oxidative Stress-Induced Loss of Self-Renewal Capacity and Stemness in Human Tendon Stem Cells by Reducing ROS Accumulation. Cell Physiol Biochem, 39:2227-2238. 10.1159/000447916 27832632

[101] Nie D, Zhang J, Zhou Y, Sun J, Wang W, Wang JH (2021). Rapamycin Treatment of Tendon Stem/Progenitor Cells Reduces Cellular Senescence by Upregulating Autophagy. Stem Cells Int, 2021:6638249. 10.1155/2021/6638249 PMC7870298 33603790

[102] Dai G, Li Y, Zhang M, Lu P, Zhang Y, Wang H, et al. (2023). The Regulation of the AMPK/mTOR Axis Mitigates Tendon Stem/Progenitor Cell Senescence and Delays Tendon Aging. Stem Cell Rev Rep, 19:1492-1506. 10.1007/s12015-023-10526-0 36917311

[103] Waddell JN, Zhang P, Wen Y, Gupta SK, Yevtodiyenko A, Schmidt JV, et al. (2010). Dlk1 is necessary for proper skeletal muscle development and regeneration. PLoS One, 5:e15055. 10.1371/journal.pone.0015055 PMC2993959 21124733

[104] Kang X, Yang MY, Shi YX, Xie MM, Zhu M, Zheng XL, et al. (2018). Interleukin-15 facilitates muscle regeneration through modulation of fibro/adipogenic progenitors. Cell Commun Signal, 16:42. 10.1186/s12964-018-0251-0 PMC6053744 30029643

[105] Gonzalez D, Contreras O, Rebolledo DL, Espinoza JP, van Zundert B, Brandan E (2017). ALS skeletal muscle shows enhanced TGF-β signaling, fibrosis and induction of fibro/adipogenic progenitor markers. PLoS One, 12:e0177649. 10.1371/journal.pone.0177649 PMC5433732 28520806

[106] Sims NA (2016). Cell-specific paracrine actions of IL-6 family cytokines from bone, marrow and muscle that control bone formation and resorption. Int J Biochem Cell Biol, 79:14-23. 10.1016/j.biocel.2016.08.003 27497989

[107] Okabe I, Kikuchi T, Mogi M, Takeda H, Aino M, Kamiya Y, et al. (2017). IL-15 and RANKL Play a Synergistically Important Role in Osteoclastogenesis. J Cell Biochem, 118:739-747. 10.1002/jcb.25726 27608420

[108] Wu Q, Zhou X, Huang D, Ji Y, Kang F (2017). IL-6 Enhances Osteocyte-Mediated Osteoclastogenesis by Promoting JAK2 and RANKL Activity In Vitro. Cell Physiol Biochem, 41:1360-1369. 10.1159/000465455 28278513

[109] Yi L, Li Z, Jiang H, Cao Z, Liu J, Zhang X (2018). Gene Modification of Transforming Growth Factor β (TGF-β) and Interleukin 10 (IL-10) in Suppressing Mt Sonicate Induced Osteoclast Formation and Bone Absorption. Med Sci Monit, 24:5200-5207. 10.12659/MSM.909720 PMC6076426 30050032

[110] Mohamed SG, Sugiyama E, Shinoda K, Taki H, Hounoki H, Abdel-Aziz HO, et al. (2007). Interleukin-10 inhibits RANKL-mediated expression of NFATc1 in part via suppression of c-Fos and c-Jun in RAW264.7 cells and mouse bone marrow cells. Bone, 41:592-602. 10.1016/j.bone.2007.05.016 17627913

[111] Liu X, Ning AY, Chang NC, Kim H, Nissenson R, Wang L, et al. (2016). Investigating the cellular origin of rotator cuff muscle fatty infiltration and fibrosis after injury. Muscles Ligaments Tendons J, 6:6-15. 10.11138/mltj/2016.6.1.006 PMC4915463 27331027

[112] Jensen AR, Kelley BV, Mosich GM, Ariniello A, Eliasberg CD, Vu B, et al. (2018). Neer Award 2018: Platelet-derived growth factor receptor α co-expression typifies a subset of platelet-derived growth factor receptor β-positive progenitor cells that contribute to fatty degeneration and fibrosis of the murine rotator cuff. J Shoulder Elbow Surg, 27:1149-1161. 10.1016/j.jse.2018.02.040 29653843

[113] Davies MR, Liu X, Lee L, Laron D, Ning AY, Kim HT, et al. (2016). TGF-β Small Molecule Inhibitor SB431542 Reduces Rotator Cuff Muscle Fibrosis and Fatty Infiltration By Promoting Fibro/Adipogenic Progenitor Apoptosis. PLoS One, 11:e0155486. 10.1371/journal.pone.0155486 PMC4871364 27186977

[114] Lee C, Agha O, Liu M, Davies M, Bertoy L, Kim HT, et al. (2020). Rotator Cuff Fibro-Adipogenic Progenitors Demonstrate Highest Concentration, Proliferative Capacity, and Adipogenic Potential Across Muscle Groups. J Orthop Res, 38:1113-1121. 10.1002/jor.24550 PMC9262119 31799698

[115] Udagawa N, Takahashi N, Katagiri T, Tamura T, Wada S, Findlay DM, et al. (1995). Interleukin (IL)-6 induction of osteoclast differentiation depends on IL-6 receptors expressed on osteoblastic cells but not on osteoclast progenitors. J Exp Med, 182:1461-1468. 10.1084/jem.182.5.1461 PMC2192181 7595216

[116] Kaneshiro S, Ebina K, Shi K, Higuchi C, Hirao M, Okamoto M, et al. (2014). IL-6 negatively regulates osteoblast differentiation through the SHP2/MEK2 and SHP2/Akt2 pathways in vitro. J Bone Miner Metab, 32:378-392. 10.1007/s00774-013-0514-1 24122251

[117] Chen B, Shan T (2019). The role of satellite and other functional cell types in muscle repair and regeneration. J Muscle Res Cell Motil, 40:1-8. 10.1007/s10974-019-09511-3 30968305

[118] Liu M, Ng M, Phu T, Bouchareychas L, Feeley BT, Kim HT, et al. (2023). Polarized macrophages regulate fibro/adipogenic progenitor (FAP) adipogenesis through exosomes. Stem Cell Res Ther, 14:321. 10.1186/s13287-023-03555-6 PMC10631219 37936229

[119] Lemos DR, Paylor B, Chang C, Sampaio A, Underhill TM, Rossi FM (2012). Functionally convergent white adipogenic progenitors of different lineages participate in a diffused system supporting tissue regeneration. Stem Cells, 30:1152-1162. 10.1002/stem.1082 22415977

[120] Arnold L, Henry A, Poron F, Baba-Amer Y, van Rooijen N, Plonquet A, et al. (2007). Inflammatory monocytes recruited after skeletal muscle injury switch into antiinflammatory macrophages to support myogenesis. J Exp Med, 204:1057-1069. 10.1084/jem.20070075 PMC2118577 17485518

[121] Dort J, Fabre P, Molina T, Dumont NA (2019). Macrophages Are Key Regulators of Stem Cells during Skeletal Muscle Regeneration and Diseases. Stem Cells Int, 2019:4761427. 10.1155/2019/4761427 PMC6664695 31396285

[122] Lemos DR, Babaeijandaghi F, Low M, Chang CK, Lee ST, Fiore D, et al. (2015). Nilotinib reduces muscle fibrosis in chronic muscle injury by promoting TNF-mediated apoptosis of fibro/adipogenic progenitors. Nat Med, 21:786-794. 10.1038/nm.3869 26053624

[123] Vumbaca S, Giuliani G, Fiorentini V, Tortolici F, Cerquone Perpetuini A, Riccio F, et al. (2021). Characterization of the Skeletal Muscle Secretome Reveals a Role for Extracellular Vesicles and IL1α/IL1β in Restricting Fibro/Adipogenic Progenitor Adipogenesis. Biomolecules, 11. 10.3390/biom11081171 PMC8392554 34439837

[124] Contreras O, Soliman H, Theret M, Rossi FMV, Brandan E (2020). TGF-β-driven downregulation of the transcription factor TCF7L2 affects Wnt/β-catenin signaling in PDGFRα(+) fibroblasts. J Cell Sci, 133. 10.1242/jcs.242297 32434871

[125] Contreras O, Cruz-Soca M, Theret M, Soliman H, Tung LW, Groppa E, et al. (2019). Cross-talk between TGF-β and PDGFRα signaling pathways regulates the fate of stromal fibro-adipogenic progenitors. J Cell Sci, 132. 10.1242/jcs.232157 31434718

[126] Contreras O, Rossi FM, Brandan E (2019). Adherent muscle connective tissue fibroblasts are phenotypically and biochemically equivalent to stromal fibro/adipogenic progenitors. Matrix Biol Plus, 2:100006. 10.1016/j.mbplus.2019.04.003 PMC7852197 33543006

[127] Molina T, Fabre P, Dumont NA (2021). Fibro-adipogenic progenitors in skeletal muscle homeostasis, regeneration and diseases. Open Biol, 11:210110. 10.1098/rsob.210110 PMC8651418 34875199

